# Mass production of polymer nano-wires filled with metal nano-particles

**DOI:** 10.1038/s41598-017-08153-0

**Published:** 2017-08-17

**Authors:** Nino Lomadze, Alexey Kopyshev, Matias Bargheer, Markus Wollgarten, Svetlana Santer

**Affiliations:** 1 Department of Experimental Physics, Institute of Physics and Astronomy, University of Potsdam, 14476 Potsdam, Germany; 2 Department of Ultrafast Dynamics in Condensed Matter, Institute of Physics and Astronomy, University of Potsdam, 14476 Potsdam, Germany; 3 Helmholtz Zentrum Berlin für Materialien und Energie GmbH, Department Nanoscale Structures and Microscopic Analysis, Hahn-Meitner-Platz 1, 14109 Berlin, Germany

## Abstract

Despite the ongoing progress in nanotechnology and its applications, the development of strategies for connecting nano-scale systems to micro- or macroscale elements is hampered by the lack of structural components that have both, nano- and macroscale dimensions. The production of nano-scale wires with macroscale length is one of the most interesting challenges here. There are a lot of strategies to fabricate long nanoscopic stripes made of metals, polymers or ceramics but none is suitable for mass production of ordered and dense arrangements of wires at large numbers. In this paper, we report on a technique for producing arrays of ordered, flexible and free-standing polymer nano-wires filled with different types of nano-particles. The process utilizes the strong response of photosensitive polymer brushes to irradiation with UV-interference patterns, resulting in a substantial mass redistribution of the polymer material along with local rupturing of polymer chains. The chains can wind up in wires of nano-scale thickness and a length of up to several centimeters. When dispersing nano-particles within the film, the final arrangement is similar to a core-shell geometry with mainly nano-particles found in the core region and the polymer forming a dielectric jacket.

## Introduction

The ongoing minimization of functional elements in nanoscience and nanotechnology raises considerable challenges of connecting them to macroscopic components. It would be highly desirable if one could dispose of wires with nano-scale cross section but that extend to macroscale dimensions without flaws until they ultimately connect to a macroscale contact or patch, which can be easily accessed^1^. For the above problem one might immediately think of e-beam lithography as one (albeit expensive) solution for purely metallic wires, but what is in fact most interesting for electronic^2–4^, optoelectronic^5–7^, electrochemical^8–10^ and information processing industry^11–13^ are *hybrid* materials such as inorganic materials/nanoparticles integrated into soft polymeric matrices. There are currently several methods to produce hybrid nano-wires ranging from simple procedures of molecular self-assembly to specialized synthetic routes. In the first case the specific interactions such as hydrogen bonding, electrostatic forces or π−π stacking can lead to self-organization of cyclic peptides, polypeptides, amino acids, rigid organic molecules, conjugated polymers and polyelectrolytes to nanofibers and nanowires^14–20^. One can also synthesize multicomponent rod-like structures that contain metals, inorganic semiconductors, and conducting polymers via template assisted *in situ* electrochemical deposition^21, 22^. Using a self-assembly approach of mesoscopic metal-polymer amphiphiles it was also possible to organize them into curved structures^23, 24^. In all above mentioned approaches the alignment and simultaneous connection to macroscopic components seems to still be out of reach.

With our approach, the above mentioned problems could at least partly be bypassed. With our discovery, how nano-wires can be produced in a self-organized manner, we can provide a simple, fast and cheap method to fabricate a wide variety of different nano-fiber systems in mass production. At the same time, the problem of achieving a very high aspect ratio is solved, and the dielectric cover of the wires is provided automatically, and can possibly be influenced separately by a simple choice of process parameters.

At the heart of our fabrication process is the careful synthesis and design of special photosensitive polymer brushes. These are thin polymer films where polymer chains are constrained by covalent bonding to a solid substrate^25, 26^. The mutual interaction between chains makes them stretch away from the surface, resulting in a brush geometry. They can be employed in a range of practical applications from tuning wettability and reducing friction to controlling adsorption of proteins and biological systems^27^. The synthesis and prediction of properties of sufficiently simple brush systems are by now well understood^28–31^. The basis of the polymer films used in our work is also a simple brush system that is, however, modified by photosensitive side chains containing azobenzene molecules^32, 33^.

Azobenzene is known as a molecular actuator that converts optical energy into mechanical work. Under irradiation with UV light the azobenzene undergoes reversible photo-isomerization from the *trans-* to the *cis-* conformation^34^. The photo-isomerization is associated with significant alteration of the azobenzene properties: the length of the molecules changes by almost a factor of two, the dipole moment changes from 0 Debye for the *trans-* (the more stable configuration) to ~3 Debye for the *cis-*isomer. When azobenzene molecules are attached to a polymer, they act as active elements that transform optical energy through molecular mechanisms to the global deformation of the polymer matrix^35^. Among the many unexpected physical phenomena related to azobenzene containing polymers is the formation of so-called surface relief gratings (SRG) upon irradiation with an interference pattern of UV light^36, 37^. During this process the polymer film deforms according to the distribution of intensity or a polarization pattern^38–42^. Opto-mechanical forces generated during the absorption of UV light are strong enough in order not only to deform glassy polymer film, but also to rupture metal films and stretch graphene multilayers deposited on top of the polymer film^43, 44^. In polymer brushes, the influence of the photosensitive side chains led to the phenomenon of opto-mechanical scission of the covalently bound chains^45–47^. The polymers are ruptured locally during the deformation process at the place where the topography minima are formed. We have used this property for instance, in order to pattern brushes on a nano-meter scale^47^. Indeed, irradiating the brush with an interference pattern of UV light, most of the polymer material after treatment with good solvent is removed from the surface, leaving characteristic line or dot patterns behind^46^.

Here we report on how one can use the process of the local rupturing of polymer chains within photosensitive polymer brushes in order to produce large amounts of ordered wires of nano-scale thickness and a length of up to several centimeters filled with nano-particles of different nature.

## Results and Discussion

**Figure 1:**
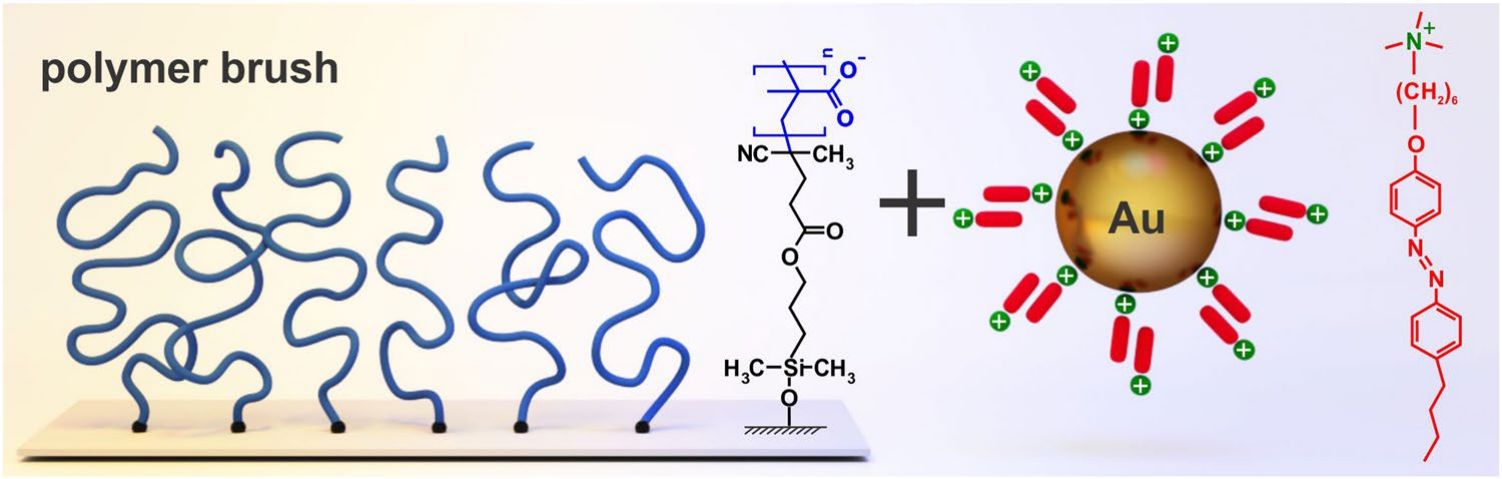
Scheme and chemical structure of the PMAA polyelectrolyte brush (blue) and cationic azobenzene containing surfactant (red). Negatively charged gold nano-particles are mixed first with cationic surfactant followed by adsorption within the brush.

In our study we utilize a poly(methacrylic acid) (PMAA) brush (prepared as described in the Material Section) with the following molecular parameters: the initial thickness in the dry state was h = 84 nm, molecular weight M_n_ = 3.1·10^6^ g/mol, and grafting density σ = 0.018 nm^−2^ resulting in a distance between neighboring chains of D = 7.4 nm (Fig. 1). The parameters of the brush were determined as described in ref. 29. The PMAA brush is negatively charged. To integrate negatively charged gold nano-particles (AuNPs) into the negatively charged polyelectrolyte brush and to simultaneously load the film with photosensitive azobenzene containing cationic surfactants (Fig. 1), we first prepare a mixture of water suspension of AuNPs (d = 10 nm) with surfactant solution in different ratios, followed by exposing the PMAA brush for 30 minutes to this solution.

**Figure 2:**
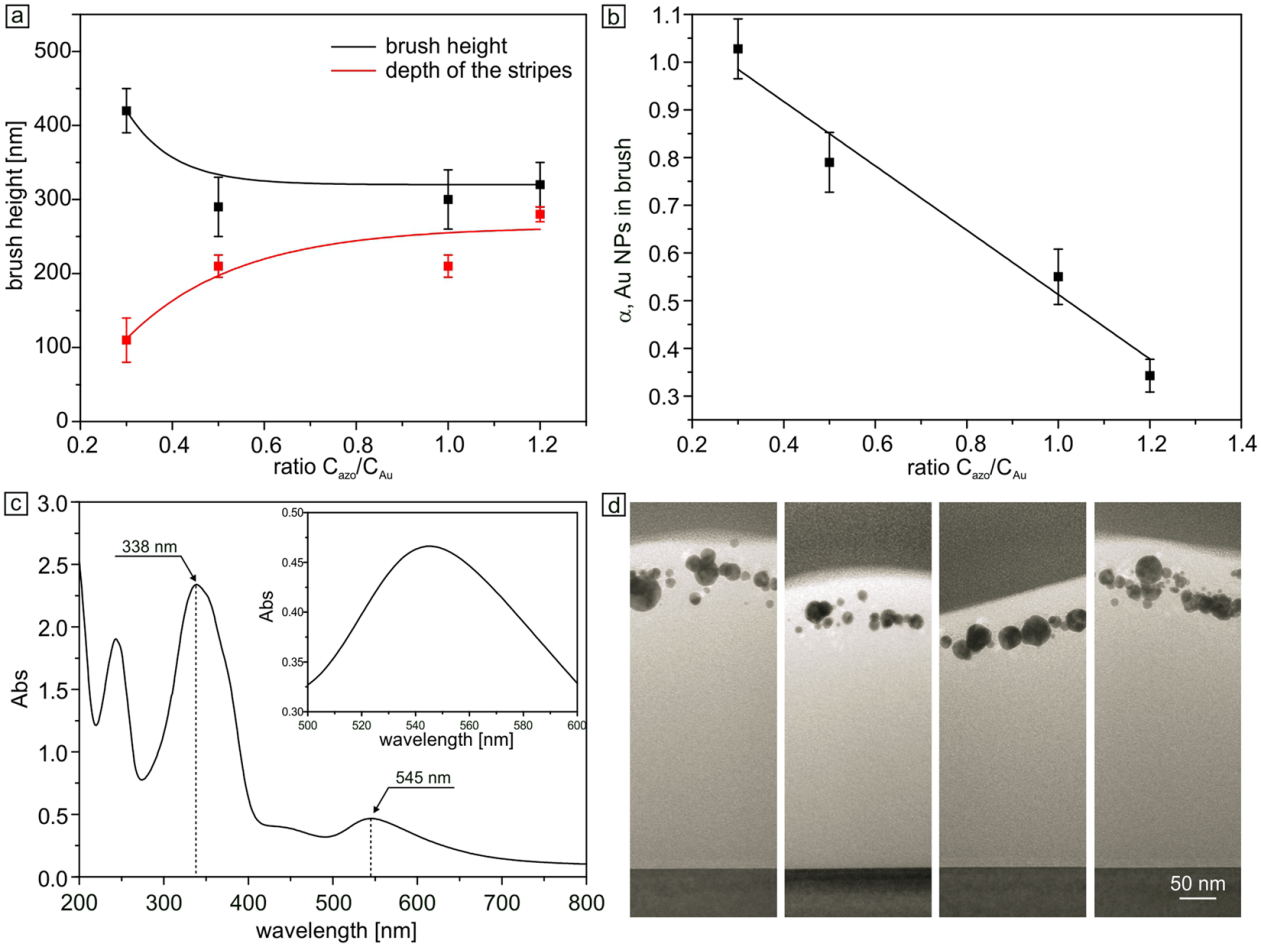
(**a**) Dependence of the height of the PMAA brush after absorption of azo-AuNPs, as a function of C_azo_/C_Au_ ratio (black curve). The red curve shows the height of the nano-wires produced from the brushes in (**b**). (**b**) Dependence of the amount of gold nanoparticles within the brush as a function of C_azo_/C_Au_. (**c**) Absorption spectrum of the PMAA brush loaded with azo-AuNPs. The inset depicts a surface plasmon peak of the gold aggregates at λ  = 545 nm. (**d**) TEM cross sectional images of the brush film obtained by cutting it using a FIB.

We chose the concentrations of the AuNPs and surfactants such that a double layer of surfactant molecules is formed around the gold particles, with charged groups pointing outside (Fig. 1)^48^. In this way, the complex of the Azo-AuNPs is positively charged, allowing for ionic interaction with the negatively charged polymer brush. When the brush was exposed to the azobenzene modified AuNPs, these particles diffuse into the brush and adsorb to the polymer matrix resulting in an increase of the brush dry thickness. It was recently reported that monovalent as well as multivalent ions cause shrinkage in swollen polyelectrolyte brushes^49–51^. In our case the increase in the brush dry thickness is explained by increase of the overall molecular weight of the polymer chains when surfactant and Azo-AuNPs complex are loaded in the film. The increase in dry thickness of the brush depends on the ratio, C_azo_/C_Au_, where C_azo_ and C_Au_ are the molar concentrations of the azobenezene containing cationic surfactant and gold nano-particles, respectively (Fig. 2a). In our study we use four solutions with ratios of C_azo_/C_Au_ equal to 0.3, 0.5, 1 and 1.2. To vary C_azo_/C_Au_ we change the concentration of surfactant while keeping the concentration of gold particles constant at C_Au_ = 1 mM. In all four cases there is a formation of an azobenzene containing shell around the AuNPs rendering them positively charged as determined by measuring the surface potential of the particles^48^. Larger values of C_azo_/C_Au_ indicate an excess of surfactant in the system. The reason for using different concentrations is to find the optimal conditions at which one can load the brush with the maximum number of AuNPs while keeping the brush photosensitive. As can be inferred from Fig. 2a (black curve), the brush height increases up to (300 ± 50) nm after absorption of azo-AuNPs for C_azo_/C_Au_ starting from 0.3. For higher surfactant concentrations, the brush thickness increases even more up to 420 nm, i.e. 5 times of its initial thickness.

Since the absorption peaks of azobenzene and Au nano-particles are well separated (λ_azo_ = 338 nm^48^, λ_Au_ = 545 nm, Fig. 2c), we determine the amount of gold nano-particles absorbed within the brush using absorption spectra of the azo-AuNPs solution before and after immobilization. Figure 2c shows corresponding spectrum of brush loaded with azo-AuNPs. The absorption peak of the surfactant is clearly observed in Fig. 2c and can be used to calculate the surfactant concentration within the brush. The surface plasmon absorption peak of the gold nano-particles within the brush is shifted to 545 nm due to the change in refractive index with respect to the water solution^52^. Figure 2b shows the dependence of the amount of gold nano-particles within the brush, α, as a function of C_azo_/C_Au_. α is the fraction of the brush surface area eclipsed by projecting the cross-sectional area of the nano-particles onto it. For instance, α  =  1 means that if the gold nano-particles would form a monolayer within the brush, the coverage of this monolayer would be 100%.

With increasing the C_azo_/C_Au_ ratio the number of absorbed gold nano-particles decreases by almost a factor of three (Fig. 2b), while the amount of adsorbed surfactant increases. To compare the projected concentration of particles to the volume concentration, we calculated also the number of nano-particles contained within the volume of a cube of 100 nm side length. For instance, for brush with maximum particle concentration one can find 160 nano-particles within a volume of 10^6^ nm^3^. With a particle diameter of 10 nm, one can estimate that the particles occupy 8% of the brush volume. This seems to be quite a low value, however, the fact that the bulky particles are not homogeneously distributed within the brush and only slightly penetrate it results in the formation of a dense layer of nano-particles not far away from the brush/air interface (Fig. 2d). In general, we find that the particles are distributed within a slab of thickness equal to roughly a fourth of the brush height (and adjacent to the air-brush interface), as can be inferred from the TEM micrograph of the brush film cut by the FIB (Fig. 2d). These results will be further supported by the SEM experiments in the following.

**Figure 3:**
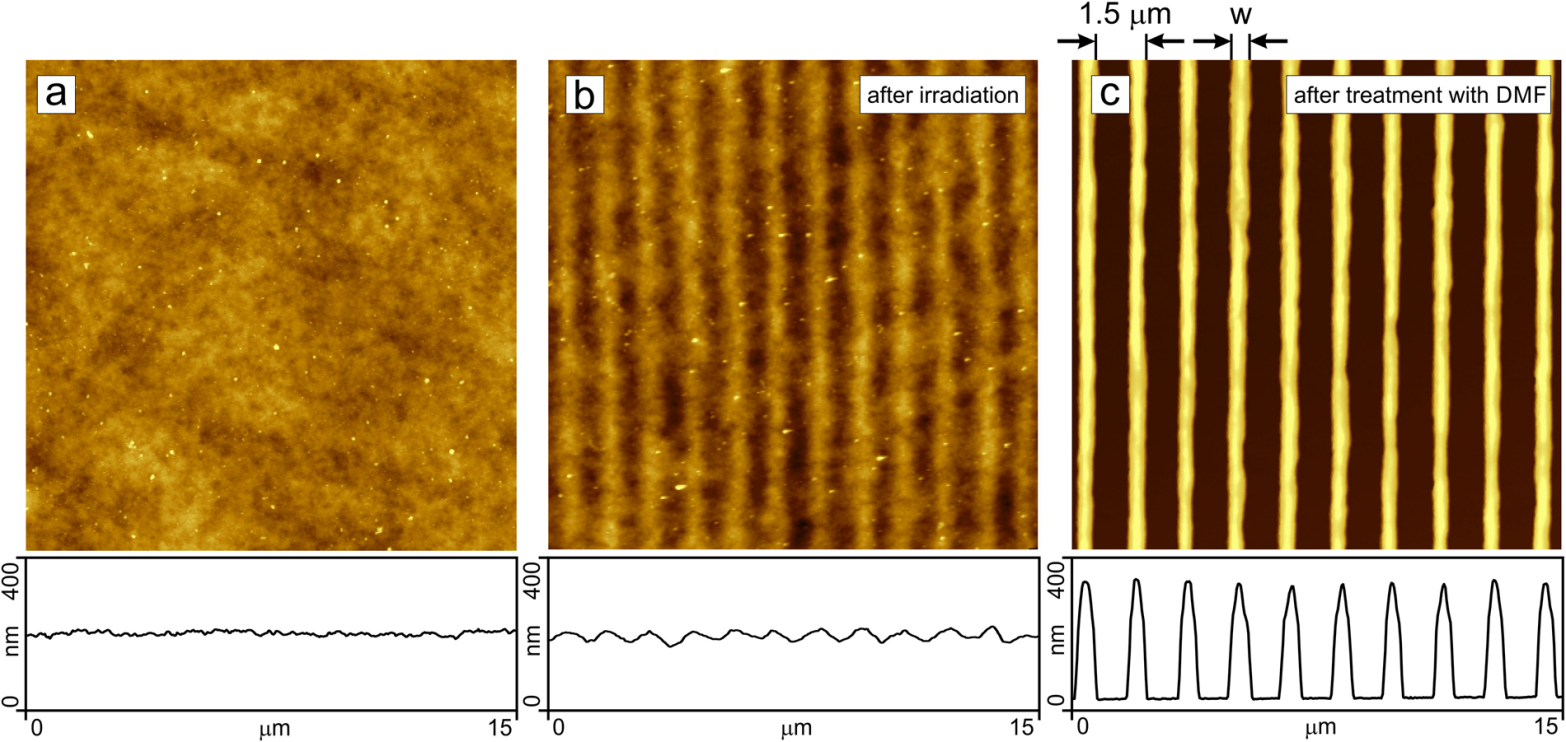
AFM micrographs of the PMAA brush loaded with azo-AuNPs before irradiation (**a**), after irradiation during 10 minutes with an IP at λ  = 325 nm (**b**), and after subsequent treatment with good solvent (DMF) (**c**). The corresponding cross-sections are shown below.

After loading the brush with azo-AuNPs its topography is still flat (Fig. 3a). Here we show the results with a brush containing the maximum concentration of gold particles. One can still see a small number of gold nano-particles (white spots in Fig. 3a and b) of 2–3 nm in height that are partially embedded in the brush but still covered with polymer material as could be inferred from a measurement of the adhesion force by means of AFM. During irradiation (E = 12.4 J/cm^2^) with an interference pattern (IP) generated of cross-propagating beams of UV light (λ  = 325 nm) a surface relief grating (SRG) forms (Fig. 3b). The height and the periodicity of the stripes are (25 ± 5) nm and 1.5 μm, respectively. We have previously shown^45–47, 53^ that during SRG formation the polymer chains rupture within areas where the polymer material is receding from, i.e. at the minima of the topography. To check whether chain scission also occurs in brushes loaded with gold nano-particles we exposed the irradiated brush to DMF which is a good solvent for both, the surfactant and the PMAA. Figure 3c shows the same area as in Fig. 3b after DMF treatment. An array of polymer stripes of w = 450 nm in width and h = 300 nm in height remains. In between the stripes one can find a thin layer of polymer material of only 5 nm in thickness. Apparently, during irradiation part of the polymer chains are ruptured and during the treatment with DMF, the ruptured chains are removed leaving behind the nano-bush like structure. The height of the stripes formed is smaller than the initial brush height. Exposing the non-irradiated photosensitive polymer brush to a good solvent results in a partial desorption of the surfactant molecules. As a consequence, the height of the brush decreases, in the present case from 400 nm to 300 nm.

**Figure 4:**
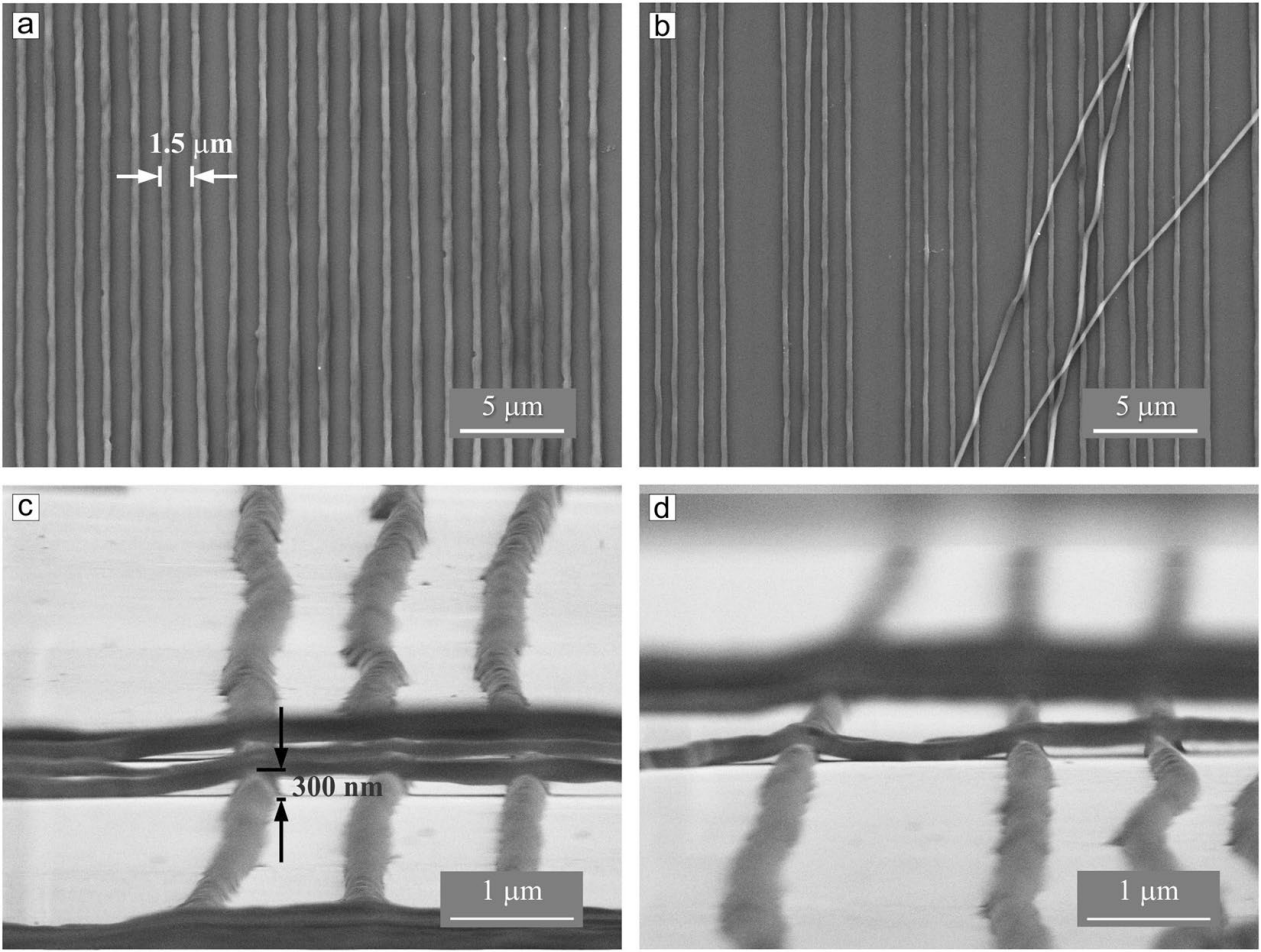
SEM micrographs of the nano-wires: (**a**) an array of 20 nano-wires perfectly aligned; (**b**) several nano-wires displaced from their initial positions by capillary forces during solvent exposure. (**c** and **d**) Side-view SEM images show clearly that the nano-wires are free standing separate entities adsorbed on a silicon surface.

**Figure 5:**
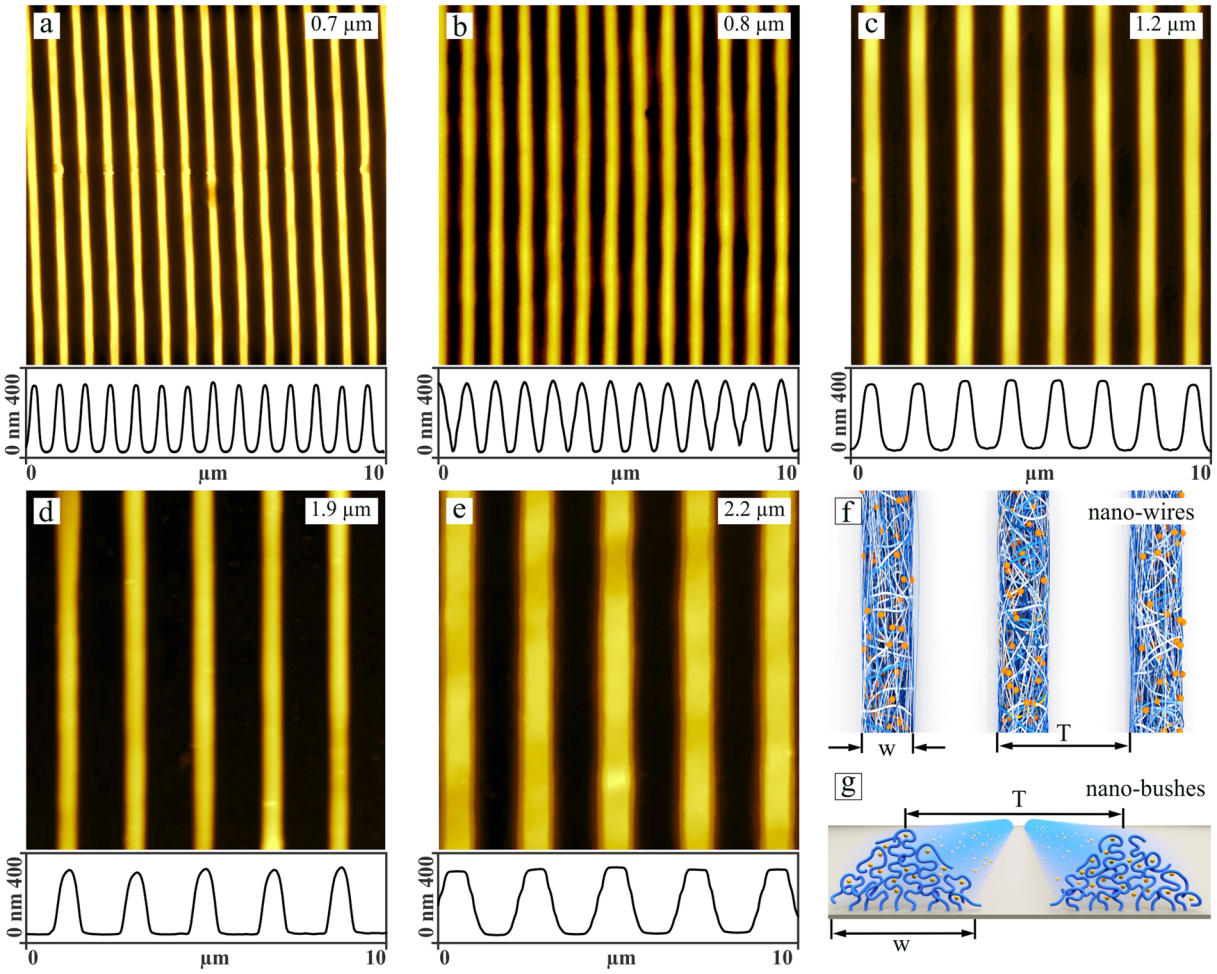
AFM micrographs of the polymer nano-wires filled with gold nano-particles (**a**–**d**). Four different periodicities and diameter of the nano-wires were obtained by adjusting the periodicity of the optical interference pattern. (**e**) At larger periodicity, not all polymer chains are ruptured, but a small amount stays still bound to a surface forming “nano-bushes” filled with gold nano-particles. (**f**) Scheme of the nano-wires, which can be formed during irradiation with polarization interference pattern. (**g**) Scheme of the “nano-bushes”, i.e. nano-structured polymer brushes. In both scheme yellow sports represent AuNP.

Applying irradiation with larger intensity, we were able to produce nano-wire like structures, i.e. stripes which are not covalently connected (i.e. free standing) to a surface. Indeed, as can be seen in Fig. 4 nano-wires are displaced from their initial position just by capillary forces. The nano-wires are formed during irradiation with polarization interference pattern at irradiation intensity of 20 J/cm^2^. Starting from the periodicity of 2.5 μm the nano-bush like structure (see for schematic representation Fig. 5g) is formed even at high irradiation intensity (20 J/cm^2^).

The width and height of the nano-wires remain unchanged after exposure to DMF solution. This indicates that the nano-wires consist of the cross-linked polymer chains filled with AuNPs. The possible mechanism of cross-linking could be a recombination of two radicals of ruptured chains. During irradiation, the opto-mechanical stress generated within the brush results in scission of covalent bonds that right after scission form shorter chains with terminal free radicals. The mutual recombination of nearby radicals may lead to cross-linking within the polymer shell of the nano-wires, explaining their stability.

The width and the periodicity of the nano-wires can be varied by adjusting irradiation conditions such as optical periodicity of the interference pattern. Figure 5(a–e) show four nano-wires of the same height but differing in the width: 320 nm, 400 nm, 580 nm, 780 nm and 1.2 μm. The length of the nano-wires is controlled by the size of the laser spot and can be as large as several centimeters implying very high aspect ratio. The number of the produced nano-wires during one irradiation process is also limited only by the size of the laser spot and can be calculated as the size of the laser spot divided over the periodicity of the interference pattern, for instance, with laser spot diameter of 1 millimeter and optical periodicity of 1.5 μm one obtains in one irradiation step ca. 666 nano-wires.

**Figure 6:**
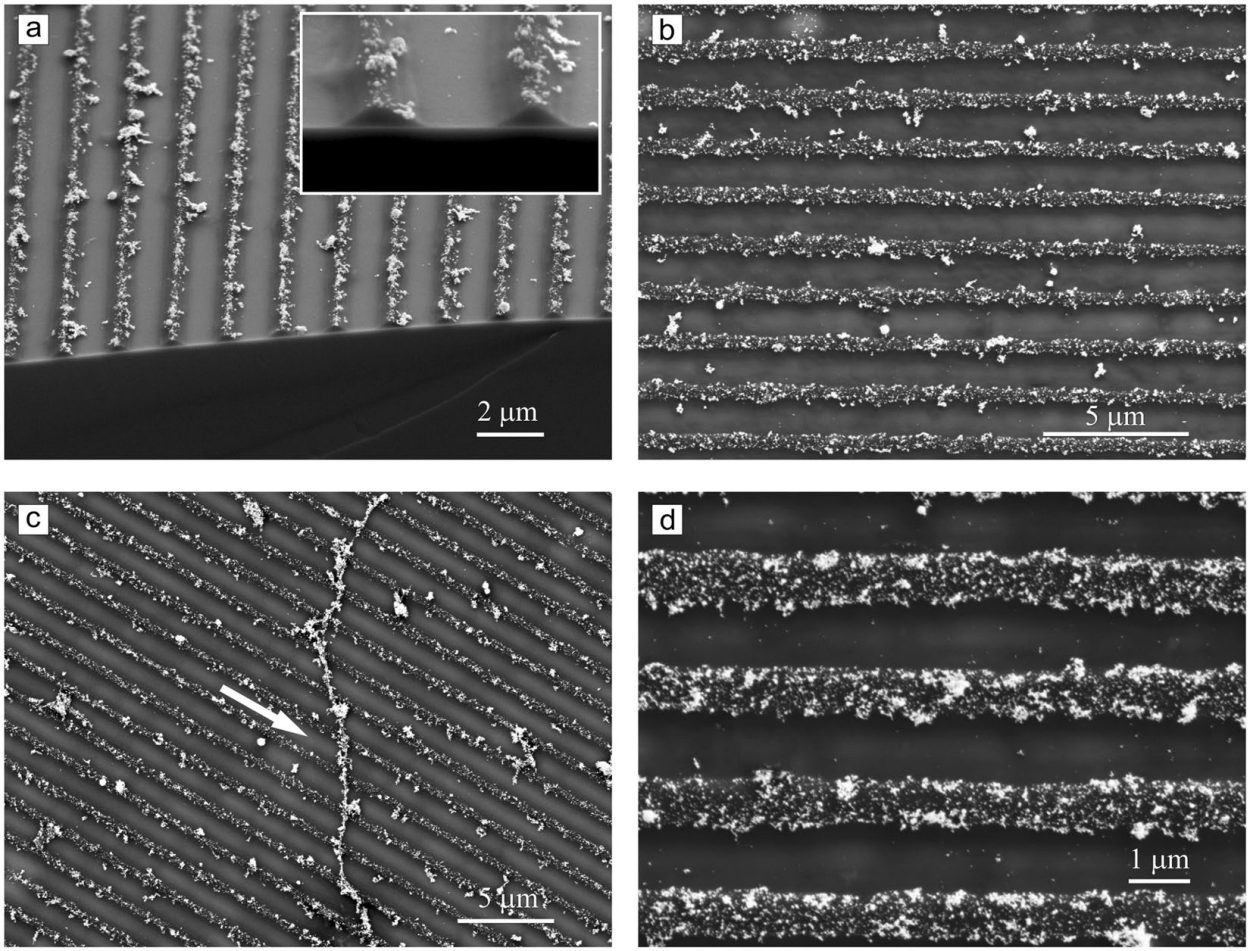
SEM micrographs of the polymer nano-wires filled with gold nano-particles. The gold nano-particles can be clearly identified within the nano-wires by their strong white contrasts. One nano-wire removed from its initial position during treatment with DMF is shown in **c** (marked by white arrow).

The nano-wires are uniform in height and width along the whole length and no gold nano-particles are found at or close to the surface of the nano-wires (Figs 3 and 4). The AuNPs are immersed within the nano-wires as can be seen in Fig. 6. To visualize gold nanoparticles within the nano-wires, we gently etch the upper polymer layer with a focused electron beam, simultaneously recording the scanning electron microscopy images. The nano-particles are distributed only within the nano-wires as a single specimen (Fig. 6d) as well as aggregates. The side view shows that the nano-particles do not penetrate the whole film and are situated in the upper fourth of the brush (Fig. 2d). The nano-wires, however, are separate independent entities with the nano-particles well embedded in the polymer, as can be inferred from Fig. 6c where one nano-wire filled with gold particles is removed from its initial position and accidentally placed by capillary forces across the others.

**Figure 7:**
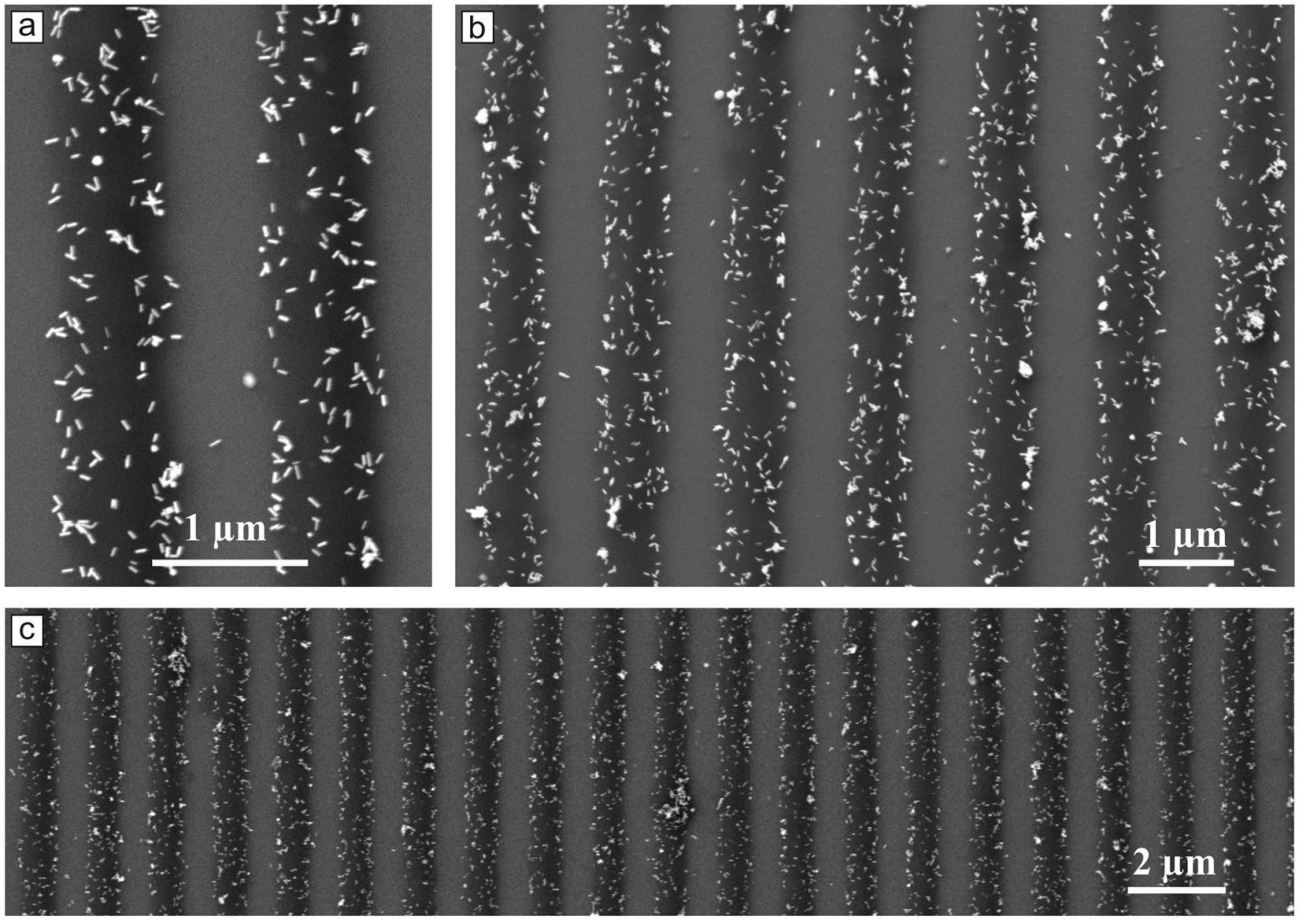
SEM micrographs of the polymer wires filled with gold nano-rods. (**a**) The gold nano-rods are randomly situated within the nano-wires. (**b** and **c**) Larger overview of the ensemble of many nano-wires.

In the very same way one can load the brush with any small molecule or particle. The requirement is that the loading substances interact with azobenzene containing surfactant and become both photosensitive and positively charged. For instance, Fig. 7 shows the polymer nano-wires loaded with gold nano-rods. The samples were prepared using the same procedure as described above.

**Figure 8:**
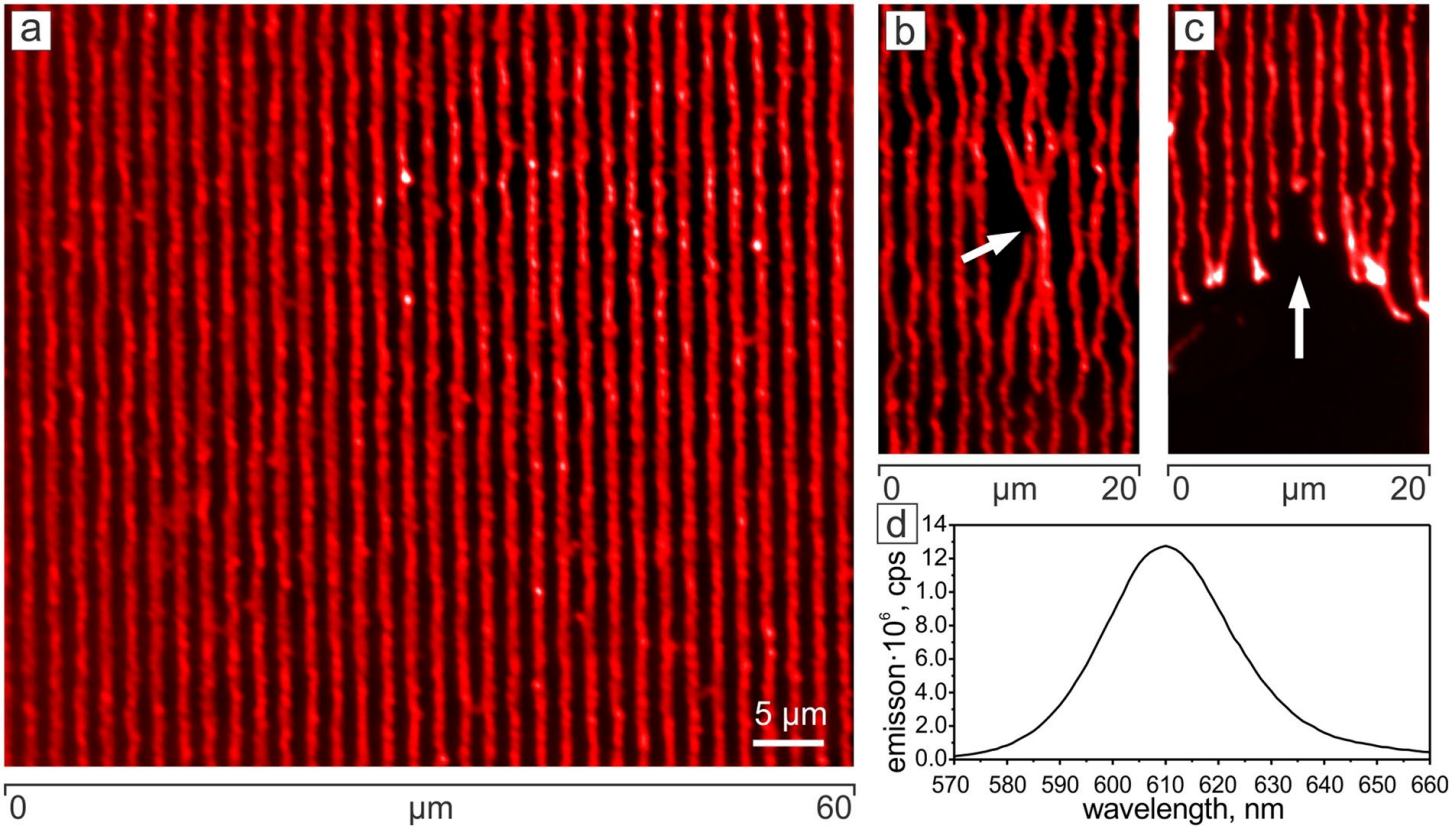
Fluorescent microscopy image of the polymer nano-wires filled with quantum dots. The QDs emit at 610 nm (see plot in d) that results in the red color of the nano-wires. (**a**) shows an ensemble of parallel ordered free standing nano-wires. (**b**,**c**) There are few defects in the ensemble where three nano-wires form a bundle at one point marked by arrow in (**b**), or they are ruptured forming a fringe as shown in (**c**).

In the next example, we have functionalized the nano-wires with quantum dots (QDs) (Fig. 8). In this case, it was possible to produce nano-wires emitting at 610 nm.

## Conclusions

We report on a mass production of spatially ordered, flexible, free-standing nano-sized wires of macroscale length filled with different types of nano-particles. The process is based on the opto-mechanical scission of chains of photosensitive polymer brushes upon irradiation with UV-interference patterns. The polymer brushes were turned photosensitive by loading with an surfactant containing azobenzene. Upon irradiating the photosensitive polymer brushes with the interference pattern, a substantial mass redistribution of the polymer material occurs resulting in a formation of surface a relief grating. Along with the polymer film deformation a rupturing of the polymer chains takes place. During treatment of the irradiated brush with a good solvent, the chains wind up in wires of nano-scale thickness and a length of up to several centimeters. We have shown that the process of nano-wire formation proceeds in the same way when nano-particles of different structure, e.g. gold nano-spheres, nano-rods, quantum dots, are dispersed within the film prior irradiation. The final arrangement is similar to a core-shell geometry, where nano-particles were mainly found in the core region and the polymer is forming a dielectric jacket.

## Materials and Methods

### Synthesis of poly(methacrylic acid) brushes

Polymethacrylic acid (PMAA) *brushes* were synthesized using a surface initiated free radical chain polymerization of methacrylic acid utilizing a “grafting from” technique on a silicon wafer^29^. PMAA chains grafted on the surface are schematically shown in Fig. 1. The brush sample was divided in several pieces of equal size, which then were loaded with different amount of nano-particles.

To integrate complexes of gold nano-particles (AuNPs), the brush was placed in a mixture of water suspension of AuNPs (d = 10 nm) with surfactant solution of different ratios for 30 minutes followed by drying with nitrogen flow.

### Synthesis and characterization of azobenzene containing surfactants

Azobenzene containing trimethylammonium bromide surfactant (Fig. 1) was synthesized as described elsewhere^54^. The surfactant was dissolved in water (MilliQ) to different initial concentrations and kept in the dark for several days to ensure complete relaxation to the trans-configuration. Then, the solutions were further diluted to an appropriate concentration for the complex preparation. All experiments were carried out under yellow light to avoid premature isomerization of the surfactant.

### Nano-particles

Gold nanoparticles (AuNPs) of diameter (10 ± 3) nm were generated by laser ablation^55^. At basic pH values the nano-particles are negatively charged as measured by a Zeta-sizer. The characteristic absorption peak of AuNPs in water originating from the excitation of surface plasmonsis is λ_SP_ = 523 nm. Quantum dots eFluor® 650 nanocrystal (CdSe) were purchased from eBioscience. Gold nano-rods were synthesized as described in ref. 56.

### Optical set-up for generation of interference patterns

The Lloyd’s mirror scheme with He-Cd laser (Kimmon) operating at λ = 325 nm (total power of ~3 mW) was used for UV interference lithography. The periodicity of the interference pattern is given by d =  λ/2sin(θ), where λ is the wavelength of the incident light, and θ is the angle between the incoming laser beam and the mirror surface. The irradiation time was 15 minutes.

### AFM measurements

Atomic Force Microscopy (AFM) (Nanoscope V, Veeco) was used to measure the height of the brushes. For thickness measurements the brushes were scratched by a glass pipette to remove the material and the step height between the top of the brush and the carried substrate were measured from the AFM cross-section analysis. *Scanning electron microscopy (SEM) measurements* were performed using a Ultraplus 4061 microscope (Zeiss). Cross sections for investigation by *transmission electron microscopy* (TEM) were prepared by cutting and thinning thin lamellae using a Ga focussed ion beam (Zeiss Crossbeam 1540 EsB). Bright field images were obtained in a Zeiss Libra 200 FE operated at 200 kV accelerating voltage. The in-column Omega type energy filter was used to exclude most of the inelastically scattered electrons to improve image contrasts (ZLF, *zero-loss filtered imaging*). *UV absorption spectroscopy*. UV-Vis spectra were obtained using a Cary 5000 UV-Vis-NIR spectrophotometer (Varian Inc.). The *Zeta-potential* was measured with a Zetasizer Nano ZS (Malvern Instruments Ltd.) at a scattering angle of 173°.
